# Evolution of Urban Haze in Greater Bangkok and Association with Local Meteorological and Synoptic Characteristics during Two Recent Haze Episodes

**DOI:** 10.3390/ijerph17249499

**Published:** 2020-12-18

**Authors:** Nishit Aman, Kasemsan Manomaiphiboon, Natchanok Pala-En, Eakkachai Kokkaew, Tassana Boonyoo, Suchart Pattaramunikul, Bikash Devkota, Chakrit Chotamonsak

**Affiliations:** 1 The Joint Graduate School of Energy and Environment, King Mongkut’s University of Technology Thonburi, Bangkok 10140, Thailand; aman.nishit@gmail.com (N.A.); eakkachai.ko@gmail.com (E.K.); devkota.bikky@gmail.com (B.D.); 2 Center of Excellence on Energy Technology and Environment, Ministry of Education, Bangkok 10140, Thailand; 3 Pollution Control Department, Ministry of Natural Resources and Environment, Bangkok 10400, Thailand; natpalaen@gmail.com; 4 Traffic and Transport Development and Research Center, King Mongkut’s University of Technology Thonburi, Bangkok 10140, Thailand; tassana.boo@gmail.com (T.B.); suchart.pat@gmail.com (S.P.); 5 Department of Geography, Chiang Mai University, Chiang Mai 50200, Thailand; chotamonsak@gmail.com

**Keywords:** urban haze, temperature inversion, Obukhov length, HYSPLIT, biomass burning, cold surge, emission

## Abstract

This present work investigates several local and synoptic meteorological aspects associated with two wintertime haze episodes in Greater Bangkok using observational data, covering synoptic patterns evolution, day-to-day and diurnal variation, dynamic stability, temperature inversion, and back-trajectories. The episodes include an elevated haze event of 16 days (14–29 January 2015) for the first episode and 8 days (19–26 December 2017) for the second episode, together with some days before and after the haze event. Daily PM_2.5_ was found to be 50 µg m^−3^ or higher over most of the days during both haze events. These haze events commonly have cold surges as the background synoptic feature to initiate or trigger haze evolution. A cold surge reached the study area before the start of each haze event, causing temperature and relative humidity to drop abruptly initially but then gradually increased as the cold surge weakened or dissipated. Wind speed was relatively high when the cold surge was active. Global radiation was generally modulated by cloud cover, which turns relatively high during each haze event because cold surge induces less cloud. Daytime dynamic stability was generally unstable along the course of each haze event, except being stable at the ending of the second haze event due to a tropical depression. In each haze event, low-level temperature inversion existed, with multiple layers seen in the beginning, effectively suppressing atmospheric dilution. Large-scale subsidence inversion aloft was also persistently present. In both episodes, PM_2.5_ showed stronger diurnality during the time of elevated haze, as compared to the pre- and post-haze periods. During the first episode, an apparent contrast of PM_2.5_ diurnality was seen between the first and second parts of the haze event with relatively low afternoon PM_2.5_ over its first part, but relatively high afternoon PM_2.5_ over its second part, possibly due to the role of secondary aerosols. PM_2.5_/PM_10_ ratio was relatively lower in the first episode because of more impact of biomass burning, which was in general agreement with back-trajectories and active fire hotspots. The second haze event, with little biomass burning in the region, was likely to be caused mainly by local anthropogenic emissions. These findings suggest a need for haze-related policymaking with an integrated approach that accounts for all important emission sectors for both particulate and gaseous precursors of secondary aerosols. Given that cold surges induce an abrupt change in local meteorology, the time window to apply control measures for haze is limited, emphasizing the need for readiness in mitigation responses and early public warning.

## 1. Introduction

Particulate matter (PM) with a size less than or equal to 2.5 µm (PM_2.5_) is an environmental concern worldwide. Suspension of such particulate matter in the atmosphere (known as aerosols) affects human health, atmospheric visibility and also impacts weather and climate both directly and indirectly [1]. The aerosols are either emitted directly in the atmosphere (known as primary sources) from combustion, wind-borne dust, sea spray, volcanic emission, and biogenic aerosol or formed in the atmosphere by conversion of primary precursor gases to secondary particles through nucleation and complex multiphase chemical reactions. High PM pollution in any place always depends upon the complex interplay between local emissions, secondary particle formation, along with local and synoptic meteorology [1,2]. Around 58% of the world population lives in areas with PM_2.5_ > 35 µg m^−3^ (in terms of daily average according to the WHO Interim Target 1) [3], many of which are highly urbanized areas or large cities. Although PM pollution episodes (sometimes known as haze episodes) are limited to few days to weeks, exposure to high PM levels even for shorter times can have an effect on human health and ecosystems [3].

Haze pollution has been studied globally to understand its formation and evolution mechanism [4,5,6,7], potentials sources contribution [5,8,9], mitigation strategies [10,11], and early warning or forecasting [12]. A haze episode may be induced by one or a number of factors combined, which encompasses emissions, secondary aerosols [13,14,15,16,17], and atmospheric transport [8,17], with unfavorable weather conditions acting as an accelerating factor [18,19,20,21,22,23,24,25]. The effects of the atmospheric boundary layer (ABL) structure, near-surface atmospheric stability, and synoptic conditions have received the attention given that they strongly dictate how haze and its associated thermal and dynamical processes evolve with time in the lower part of the troposphere [23,24,25]. Quan et al. (2013) [19] and Petaja et al. (2016) [20] suggested a positive feedback cycle for heavy air pollution where heat flux decrease significantly due to decreased solar radiation blocked by the haze layer, which in turn further decreases ABL height and trap air pollutant within. Tie et al. (2017) [21], and Liu et al. (2018) [22] reported that decreased ABL height leads to increased relative humidity, which enhances secondary aerosol formation and also promotes the hygroscopic growth of aerosols and light scattering. The latter effect reduces incident solar radiation more. Temperature inversion at different levels of the troposphere may be caused by radiation, advection, large-scale subsidence, etc. Low-level temperature inversion layers effectively reduce the atmospheric volume available for diluting air pollutants while weak winds poorly ventilate them out of a polluted area [23,24,25]. Turbulence is another important factor as the capability of mixing airborne constituents vertically in the ABL. Low turbulence, typically under stable conditions, suppresses vertical mixing, which allows pollutants and/or precursors to accumulate at near-surface levels [23,26]. Synoptic weather is another relevant aspect because it sets favorable or unfavorable background conditions to haze formation and evolution and influences local or urban-scale weather processes as well [23,24,25,26,27].

Bangkok, the capital of Thailand, and its five neighboring provinces (Samut Prakan, Samut Sakhon, Nonthaburi, Nakhon Pathom, and Pathum Thani) are collectively known as Greater Bangkok (GBK) or Bangkok Metropolitan. It is one of the largest urban agglomerations in Southeast Asia. It has experienced haze pollution typically found in the dry season, posing great concern to the general public and challenges to the local and central governments for mitigation and prevention. PM_2.5_ exceeds the daily (i.e., 24-h average) national ambient air quality standard (NAAQS) of 50 µg m^−3^ several times per year, according to the Pollution Control Department (PCD) [28], particularly in the dry season. Several PM-related studies in GBK have been conducted e.g., [29,30,31,32,33,34,35,36,37,38], see Table S1 in Supplementary Materials for details, ranging from source apportionment, chemical characterization, emission inventory, and human health. On-road vehicles (i.e., traffics) and biomass burning were identified as the major PM_2.5_ sources [29,30,31,32]. In the dry season, agricultural burning to clear crop residues on lands within GBK and its vicinity and forest fires in the northern region are generally intensified, and smoke can also be dispersed or transported to GBK [31,32,33,34]. However, biomass burning contributes little to air pollution in GBK during the summer due to a shift in the prevailing winds [35]. Pham et al. (2008) [36] estimated gaseous and particulate emissions from industrial and power plant sectors, finding the central and central regions (among all regions in Thailand) to have the largest intensities and shares. Secondary aerosols were also reported as a nonnegligible contributor [30,32]. Effects of increased PM_2.5_ on health have been emphasized and quantified [37,38]. Although these past studies provide useful information of the PM sources and PM effects for the study area in question, elevated haze and its associated meteorological dependence are still lacking or little addressed. Accordingly, this study aims to fill this knowledge gap by investigating how urban haze is influenced by meteorology at both local and synoptic scales. Specifically, we seek to understand how events of elevated haze (in terms of PM_2.5_) temporally evolve with meteorological factors using several observational datasets combined, with particular attention to cold surge as a synoptic disturbance relevant to the region and here suspected to induce favorable conditions for haze to elevate. Here, an intensive observational analysis was performed and discussed for two recent wintertime haze episodes in GBK using data from various sources to examine the association of haze with local and synoptic weather conditions.

## 2. Data and Methods

### 2.1. Study Area

Greater Bangkok (GBK) is located in the lower part of Central Thailand. It currently has a registered population of 11 million and an area of 7762 km^2^ [39]. It is the largest national hub of the economy, accounting for 46% of its total gross domestic product (GDP) [40]. It has a complex mixed (built and natural) landscape with the co-existence of commercial, residential, agricultural, and industrial areas [41]. The overall terrain of GBK is generally flat with limited heights (<10 m above mean sea level or MSL). Its general climate is tropical and humid and governed mainly by the northeast monsoon (November–February as the winter) and the southwest monsoon (May–October as the wet season) [42]. The former monsoon brings cool, dry air from continental mid-latitudes over which persistent strong high-pressure systems are present. The latter monsoon brings moist air from the Indian Ocean and the Gulf of Thailand, causing abundant rain in most parts of Thailand. The monsoon trough or intertropical convergence zone (ITCZ), which moves along the north-south direction, and tropical cyclones developed in the North Indian and Western Pacific Ocean Basins can modulate rain at a sub-seasonal scale. Importantly, a cold surge is a synoptic phenomenon, characterized by a transient southward propagation or extension of a high-pressure system from mid-latitudes (i.e., mainland China and East Asia) to the Indochina Peninsula and the equatorial South China Seas [43,44,45]. During the winter, cold surges occur episodically more often, strengthening the northeast monsoon. The arrival of a cold surge typically brings strong winds due to high-pressure gradients with an abrupt drop in temperature. Once it weakens, the cold surge recedes back or dissipates. The transitional period between the two monsoons (March–April) has relatively warm conditions, corresponding to the summer season. Here, the winter and summer combined are called the dry season [42].

### 2.2. Data

**Figure 1:**
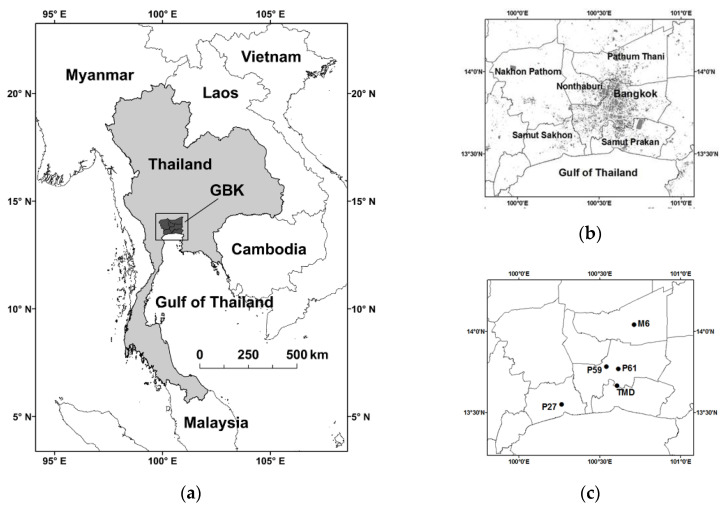
(**a**) Thailand; (**b**) Greater Bangkok and its provinces; (**c**) monitoring stations. In (**b**), the urban built-up areas (urban residential, industrial, and commercial classes combined) are shown in shaded gray, based on LDD (Land Development Department) (2016) [41]. In (**c**) P27, P59, and P61 are the air quality stations at Samut Sakhon Witthayalai School, Public Relation Department, and Bodindecha School, respectively, M6 is the 100-m tower at Techno Thani, and the Thai Meteorological Department (TMD) is the standard weather station (WMO No. 48453) at Bang Na District.

The PCD is the main government agency that administers air quality monitoring stations across Thailand. Here, hourly PM_2.5_ and PM_10_ data for the years 2015–2017 at three air quality stations (P27, P59, and P61) in the study area were requested and obtained (Table 1 and Figure 1c). P27 is located near a busy major highway (by about 70 m). P59 is in a semi-general area but not far from a busy local street and a major expressway to the west by 0.6 km. P61 is in a general area with no major road nearby within 1 km, representatively selected as the key station to support several parts of the analysis. It is noted that the PM_2.5_ data at P59 are available only from April 2015. Given missing data and discontinuity in meteorological measurements at these three stations, the 100-m tower (M6) of the PCD at Techno Thani in Pathum Thani was the main source of meteorological data for use. The tower measures air temperature (*T*) at 2 m, 50 m, 75 m, and 100 m, wind speed (*WS*) and wind direction (*WD*) at 10 m, 50 m, and 100 m, and other near-surface variables, i.e., relative humidity (*RH*), rain (*RN*) and global radiation (*GR*). PM_2.5_ and PM_10_ are real-time monitored using the standard beta-ray method at 3 m above ground level (AGL). Data are intensively quality controlled/assured by the PCD internally before distribution. The detectable limits or probable ranges of data are PM_2.5_ and PM_10_ (3 to 1000 µg m^−3^), temperature (–5 to 50 °C), relative humidity (0 to 100%), wind speed (0 to 50 ms^−1^), wind direction (0° to 360°), rain (0 to 1000 mm h^−1^) and global radiation (0 to 1000 W m^−2^) [46,47]. We applied these ranges for data screening and also removed suspicious or erratic values if found visually. Relative humidity data at the M6 tower are absent throughout 2017. Simple linear-regression extrapolation using the data at P59 was employed to gap-fill them (details not shown). The tower is in a suburban/rural area, with most of the land near or surrounding the tower being water and paddy fields. Built-up areas are also well present over its northeastern quadrant within 2 km. Since many buildings taller than 10 m are also in their proximity of 100 m, 10-m wind data were discarded. Upper-air sounding data at the Bang Na weather station of the Thai Meteorological Department (TMD) was obtained (available at http://weather.uwyo.edu/upperair/sounding.html). However, radiosonde soundings are routinely operated only at 7 local time (LT) (i.e., 0 UTC), with pilot balloons alone at 1, 13, and 19 LT. The upper-air sounding and data of the TMD typically follow the standard quality assurance of the World Meteorological Organization (WMO). Here, radiosonde-based upper-air data up to a height of 4 km were considered and extracted. To convert hourly data to a daily scale for air quality and meteorological variables, 24-h (1–24 LT) averaging was typically applied. For global radiation, a period of 11–16 LT was used to represent late-morning to mid-afternoon hours. All computations and statistical tests were performed using software R (R development core team, 2019) [48]. In any statistical calculation, 50% of data as valid/ non-missing was necessarily required as the minimum threshold.

### 2.3. Selection of Haze Episodes

**Figure 2:**
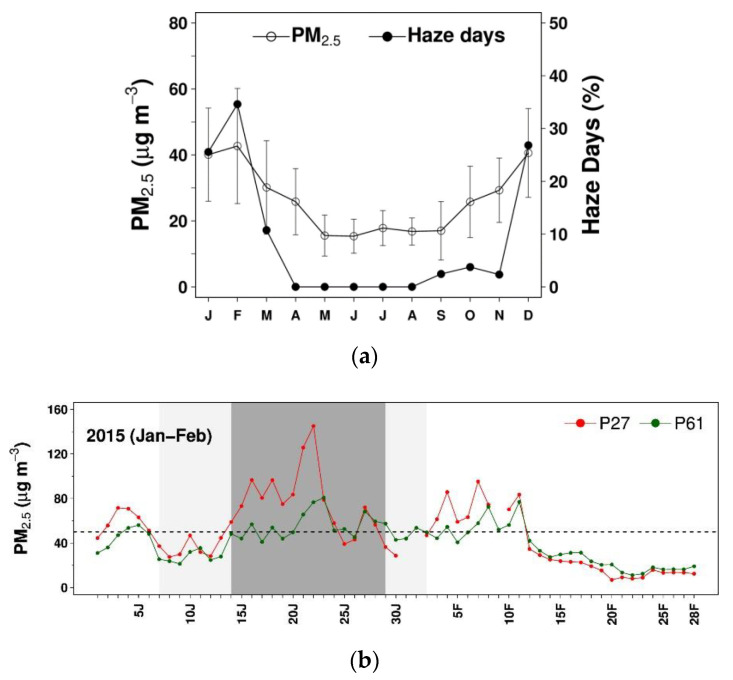

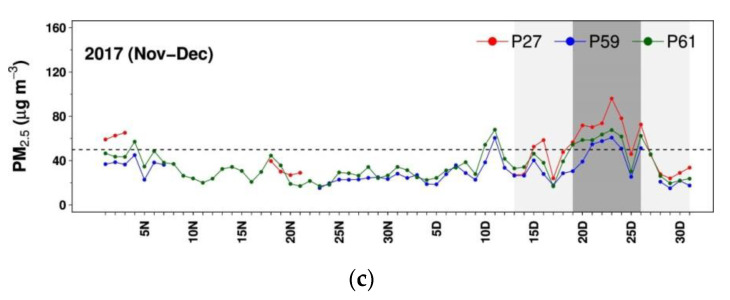
(**a**) Monthly particulate matter (PM) with a size less than or equal to 2.5 µm (PM_2.5_) and number of haze days observed at P61 over 2015–2017; (**b**) daily PM_2.5_ during haze episode 1 (EP1); (**c**) daily PM_2.5_ during haze episode 2 (EP2). In (**a**), the filled and unfilled circles are the averages, the vertical bars are the standard deviations, and the *x*-axis labels (J, F, M, …, N, and D) denote the months of the year. In (**b**,**c**), the suffixes N, D, J, and F on the *x*-axis labels correspond to November–February, respectively. In (**b**,**c**), the dark-gray shading marks the HZ periods, and the light-gray shading marks the pre-haze event (HZ) and post-HZ periods for each haze episode.

A haze day is here defined as the day with PM_2.5_ exceedance (i.e., daily PM_2.5_ level ≥ 50 µg m^−3^) registered at one station at least. Then, a haze event is simply defined as the period of consecutive haze days. As seen from Figure 2a (also see Figure S1 in Supplementary Materials), December–February is typically the period when haze intensifies. Given emphasis to extended (e.g., haze days over a week or longer) severe episodes and the amount of the valid surface and upper-air data, two haze episodes (EP1 and EP2) were representatively chosen (Figure 2b,c). In EP1, it comprises the following three periods in sequence: pre-HZ1, HZ1, and post-HZ1. The haze event (namely, HZ1) spans 16 days (14–29 January 2015). Pre-HZ1 and post-HZ1 are the periods before (7 days) and after (4 days) HZ1, respectively, when PM_2.5_ was at relatively low levels. Similarly, EP2 covers pre-HZ2, HZ2, and post-HZ2. The haze event (namely, HZ2) spans 8 days (19–26 December 2017), with 6-day pre-HZ2 and 5-day post-HZ2.

### 2.4. Synoptic Patterns

The 6-hourly 0.5°-resolution Climate Forecast System Version 2 (CFSv2) reanalysis data [49] were used to provide sea-level pressure at 13 LT for all days in EP1 and EP2 to help construct daily synoptic surface charts. Moreover, satellite infrared images (at the same local time) captured by geostationary Himawari-8 (available at http://weather.is.kochi-u.ac.jp/sat/gms.sea/) were used to examine the presence of low and high clouds over the central region of Thailand, the ITCZ, and tropical cyclones. It is noted that the constructed CFSv2-based surface charts were also compared with the corresponding TMD surface charts (available at https://www.tmd.go.th/en/weather_map.php), both of which were found to be similar. A total of four simple synoptic patterns (numbered as 0, 1, 2, and 3) were proposed here to help support the analysis, using our visual examination of the constructed charts. The representative surface charts for EP1 and EP2 are shown in Figures S2 and S3 (Supplementary Materials), respectively. The four synoptic patterns classified are as follows:

- Pattern 0: No distinct synoptic features over the Indochina. The ITCZ tends to stay over the central or lower portions of the Gulf of Thailand. On some days over GBK and the central region, clouds develop or scatter from the ITCZ edge;
- Pattern 1: A cold surge propagates southward, and its front reaching the Indochina with relatively weak pressure gradients (i.e., weak winds) over the central region. For the ITCZ and clouds, same as Pattern 0;
- Pattern 2: When the cold surge continues to propagate southward and its front reaching in the central region with moderate-to-strong pressure gradients (i.e., stronger winds). Clouds over GBK and central region are quite limited;
- Pattern 3: When the cold surge weakens over the central region, or its front recedes northward or dissipates. For the ITCZ and clouds, same as Pattern 0.

### 2.5. Temperature Inversion and Obukhov Length

A temperature inversion refers to an atmospheric condition when the air temperature increases with height, and its presence can restrict the volumetric dilution of air pollutants [2,50]. Using the radiosonde-based upper-air data (available only at 7 LT), temperature inversion layers were identified. Those only found over the heights of 100–1500 m are of interest here. Those below 100 m were excluded since they are typically induced by continuous radiative surface cooling over the nighttime and early morning hours. The upper limit of 1500 m conservatively marks the ABL thickness. A single inversion layer is given as all successive vertical levels from the sounding with temperature monotonically increasing with height. Inversion intensity (*IV*) is a simple parameter used to indicate the extent of difficulty or blockage to which air pollutants penetrate an inversion layer, which was here computed as the rate of temperature change (°C per 100 m depth) from the bottom to the top of the inversion layer, similar to Dai et al. (2020) [7]. Only inversion layers with *IV* > 0.1 °C per 100 m were considered. Obukhov length (*L*) is an important measure of near-surface dynamic stability [2,18,23], suggesting the capability of vertical mixing for air pollutants between the surface and higher levels. Its positive/negative values correspond to stable/unstable conditions. The smaller magnitude of *L*, the larger degree of stability/instability. A very large *L* in magnitude corresponds to the neutral condition. The term “dynamic” implies that both mechanical and thermal turbulence production processes are taken into account. Following the Monin–Obukhov similarity theory [18,23], *L* is computed by

(1) $$L\;=\frac{1}{2}\frac{\left(T_{v1}+T_{v2}\right)u_{*}^{2}}{kg\theta_{*}},$$

(2) $$u_{*}\;=k\;U\left(z_{u}\right){\left[ln\left(\frac{z_{u}}{z_{o}}\right)-\Psi_{M}\left(\frac{z_{u}}{L}\right)+\Psi_{M}\left(\frac{z_{0}}{L}\right)\right]}^{-1},\;\mathrm{and}$$

(3) $$\theta_{*}\;=k\;\left(\theta_{v2}-\theta_{v1}\right){\left[ln\left(\frac{z_{\theta_{v2}}}{z_{\theta_{v1}}}\right)-\Psi_{H}\left(\frac{z_{\theta_{v2}}}{L}\right)+\Psi_{H}\left(\frac{z_{\theta_{v1}}}{L}\right)\right]}^{-1},$$

where u_*_ is the frictional velocity, θ_*_ is the temperature scale, *k* is the von Karman constant (0.4), *g* is the acceleration due to gravity, *z_0_* is the roughness length, *z_u_* is the single measurement height of wind (here, 50 m or 100 m separately), *U*(z_u_) is the hourly wind speed, $z_{\theta_{v1}}$ and $z_{\theta_{v2}}$ are the two measurement heights of temperature (here, 2 m and 50 m, respectively), *θ_v1_* and *θ_v2_* are the virtual potential temperature at those two heights, *T_v1_* and *T_v2_* are the virtual temperatures at those two heights, respectively, and $\Psi_{M}$ and $\Psi_{H}$ are the Businger stability correction functions for wind and temperature, respectively. As in Kamma et al. (2020) [51], the surrounding area of the M6 tower within a 2 km radius was assessed using Google Earth (https://www.google.com/earth/) (Google, Mountain View, CA, USA) and visually examined, finding ponds and paddy fields being dominant with built-up areas present in its northeast quadrant. Using the classification by Stewart and Oke (2012) [52], the approximate local climate zone is “sparsely built” with terrain roughness (or Davenport) class 5, whose roughness length (*z_0_*) equals 0.25. The concept of virtual (potential) temperature is necessary for humidity correction, which was implemented using the “aiRthermo” package in R [53]. In doing so, surface pressure data were required, extracted from 3-hourly 0.25°-resolution global land data assimilation system (GLDAS) [54] (available at https://giovanni.gsfc.nasa.gov/giovanni/), and then linearly interpolated to an hourly scale. If pressure at any higher levels was needed, the hydrostatic adjustment was applied. We attempted to compute hourly *L* with *z_u_* = 50 m and *z_u_* = 100 m separately and found a very high correlation (0.99) between the results from the two cases. Thus, the average *L* values over both cases were used.

### 2.6. Back-Trajectories

To investigate the potential transport of air pollutants from nearby and far areas [23,24], daily kinematic back-trajectories were simulated for all days in both episodes (EP1 and EP2) by the Hybrid Single-Particle Lagrangian Integrated Trajectory model (HYSPLIT) of the National Oceanic and Atmospheric Administration (NOAA) [55]. Each back-trajectory starts at 13 LT (as a typical midday time with developed ABL) and at 500 m AGL (as a typical mid-ABL height) and migrates backward in time for 48 h. HYSPLIT was run online (at https://www.ready.noaa.gov/HYSPLIT.php) using hourly 0.5°-resolution global data assimilation system (GDAS) data for driving wind fields. Given that biomass burning (agricultural burning and forest fires) in Upper Southeast Asia is well present in the dry season [32,33,34], daily 1-km active fire hotspots detected by the MODIS (Moderate Resolution Imaging Spectroradiometer) sensors onboard of both Terra and Aqua satellites (MCD14ML Collection 6) [56] (available at https://firms.modaps.eosdis.nasa.gov/download/) were downloaded for both episodes. The fire hotspots were then summed and gridded to 0.5° according to the pre-HZ, HZ, and post-HZ periods for each episode.

## 3. Results and Discussion

The urban haze in GBK is generally associated with multiple factors and their interdependence or interplays, ranging from emissions from local sources and biomass burning, mid- and long-range transport, secondary aerosols, and meteorological conditions at both local and synoptic scales. Here, the last factor is our main focus, for which general and distinct meteorological features and how they are coupled with the urban haze evolving during the selected two haze episodes are described.

### 3.1. Haze Episode EP1

**Figure 3:**
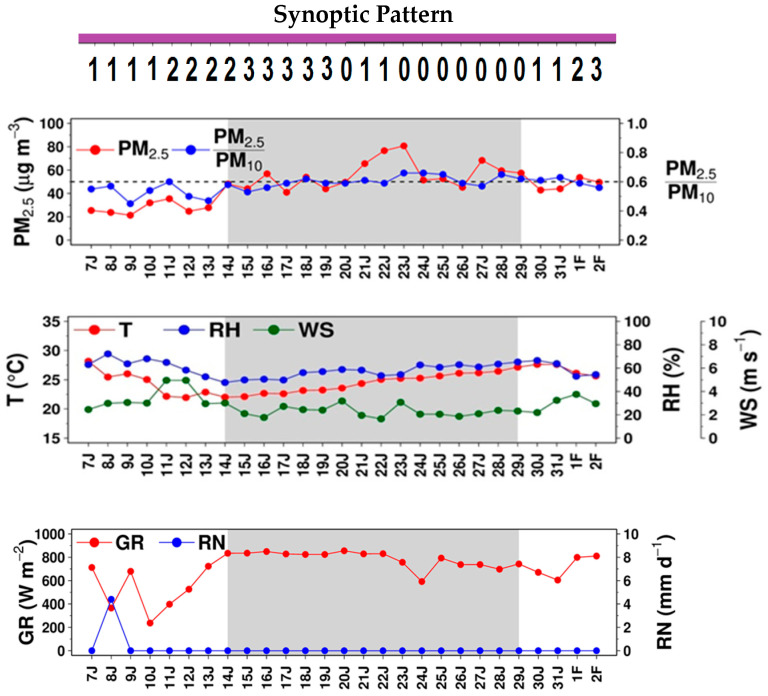
Daily PM_2.5_, PM_2.5_/PM_10_ (at station P61) and other meteorological variables during EP1.

This episode (EP1) occurred mostly in January 2015, and the haze event (HZ1) spans14–29 January. Figure 3 displays the day-to-day variation of PM_2.5_, PM_2.5_/PM_10_ ratio, temperature, relative humidity, wind speed, global radiation, rain, and synoptic pattern in the episode. Every variable was of 24-h average, except for global radiation (11–16 LT). For PM_2.5_/PM_10_, its daily values were of the 24-h average of the ratio of hourly PM_2.5_ to hourly PM_10_. As seen from the figure, PM_2.5_ was relatively high in HZ1 but low during both pre-HZ1 and post-HZ1. It showed two peaks, 80.7 µg m^−3^ on 23 January and 68.3 µg m^−3^ on 27 January. PM_2.5_/PM_10_ did not appear to vary much (ranging between 0.45 and 0.66), being slightly higher in HZ1 than pre-HZ1 and post-HZ1, suggesting fine PM mode to increase when the haze was more developed. Temperature and relative humidity were 28.2 °C and 62.9%, at the start of EP1, respectively. Both decreased to 22.0 °C and 47.6%, respectively, at the start of HZ1, as caused by the synoptic change from Pattern 1 to Pattern 2 (i.e., cold surge reaching GBK with cool, dry air), but later climbed up continuously until HZ1 ended, corresponding synoptically to the weakening cold surge or its eventual dissipation. Winds appeared to follow the synoptic patterns, which were relatively strong during the cold surge arrival (i.e., Pattern 2), as seen on 11–14 January and became weaker on the other days, particularly during HZ1, supporting the buildup of haze.

Global radiation was relatively low in pre-HZ1 (with the minimum of 237.1 W m^−2^) but turns relatively large in HZ1 (with the maximum of 849.4 W m^−2^), which could be attributed partly to fewer clouds induced by the high-pressure cold surge (i.e., shifted synoptic patterns). No rain was observed over the entire episode, except on one single day in pre-HZ1 with a light amount (4.4 mm).

In view of diurnal variation (Figure S4 in Supplementary Materials), during pre-HZ1, PM_2.5_ was generally low (<30 µg m^−3^ for most of the hours). Once the haze sets in, PM_2.5_ showed diurnality more clearly, i.e., relatively low in the afternoon but high in the nighttime and morning. Furthermore, we noticed that diurnal variation in the first (14–23 January) and second (24–29 January) parts of HZ1 showed a sharp contrast and PM_2.5_ became relatively high in the afternoon in second part as compared to the afternoon PM_2.5_ in the first part. We looked to daytime Obukhov length but did not find any dramatic change in instability over the days at all (Figure S4 in Supplementary Materials). Hence, it was not possible to explain it directly, and we then suspected that secondary aerosols were enriched in the afternoon for the later part of HZ1, given that this haze event extended as long as 16 days with increasing temperature and humidity and ample global radiation.

**Figure 4:**
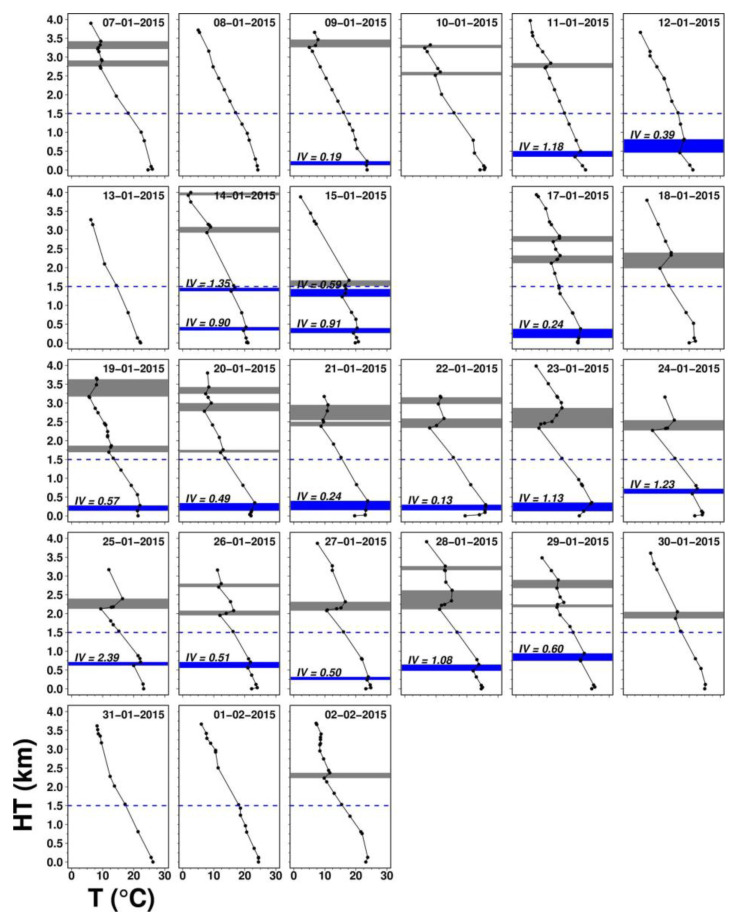
Daily vertical temperature profiles at 7 local time (LT) during EP1. The blue rectangles are the low-level inversion layers (found at 0.1–1.5 km), while the gray rectangles are the upper-level ones (found at 1.5–4 km). In the figure, *IV* represents the inversion intensity in °C/100 m.

Figure 4 shows daily vertical temperature profiles with inversion layers identified. Low-level inversion occurred for 4 days (out of 7) during pre-HZ1 but persistently appeared almost every day (14 out of 15 days with non-missing data) in HZ1. The latter highlights the limited diluting volume for air pollutants and facilitate PM_2.5_ elevation. Inversion intensity varied day-to-day during HZ1, with a minimum of 0.13 °C/100 m on 22 January and a maximum of 2.39 °C/100 m on 25 January. As mentioned previously, the temperature was relatively low after the cold surge arrives, and before it receded or dissipated, the low-level inversion was more easily induced. Even though global radiation and near-surface dynamic instability were present, it still took more heat or longer time to warm the surface to break up the inversion, based on the concept of the bulk model of daytime mixing height [47]. With a sequence of inversion-breakup failures, multiple low-level inversion layers could form, which was seen twice in HZ1. Atmospheric aerosols were known for radiative effects, e.g., black carbon or soot to absorb heat and sulfate to scatter radiation, and they could play a role in modifying temperature profiles. However, this subject is beyond the scope of the current study. Upper-level inversion also existed before the cold surge arrival and maintained over most of EP1, suggesting the presence of large-scale subsidence inversion aloft.

**Figure 5:**
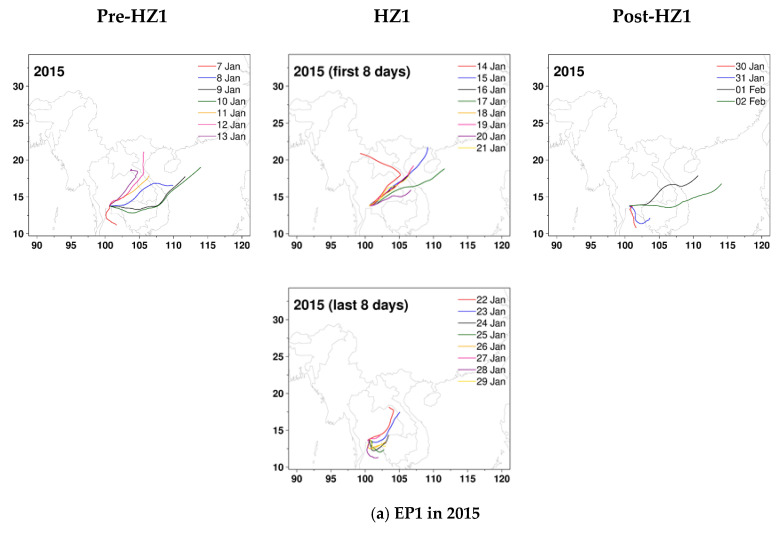

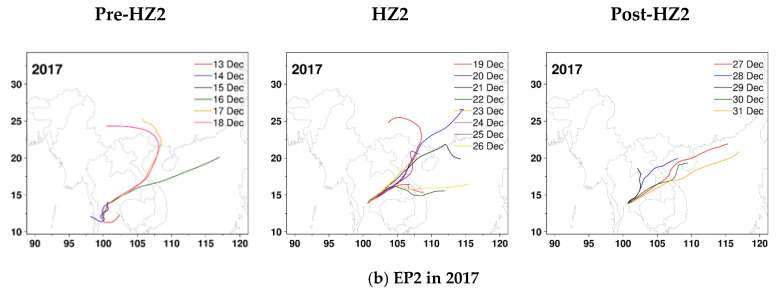
Daily 48-h back-trajectories: (**a**) EP1 in 2015; (**b**) EP2 in 2017.

Lastly, the transport of air pollutants was investigated using back-trajectories (Figure 5 and Figure S5 in Supplementary Materials). Over the course of EP1, most of the back-trajectories moved from the eastern and northeastern directions, allowing traveling air masses to absorb and carry air pollutants or emissions to the study area. Coincidentally, biomass burning was present in Laos, Cambodia and the central and northeastern regions of Thailand and became relatively intense during HZ1. Only two air masses (out of 7) pass through fire areas in pre-HZ1, while all air masses move over such areas during HZ1 (Figure S6 in Supplementary Materials). Slow-moving and low-level back-trajectories found in the second part of HZ1 (24–29 January) possibly worsened the PM_2.5_ situation in the study area (Figure S5 in Supplementary Materials). During post-HZ1, half of the back-trajectories (2 out of 4) were maritime (i.e., originating in or passing over the Gulf of Thailand) and thus relatively clean. It is, thus, fair to say that, besides local and synoptic meteorology, the long-range transport potentially impacted the urban haze in GBK to a certain extent in this episode.

### 3.2. Haze Episode EP2

**Figure 6:**
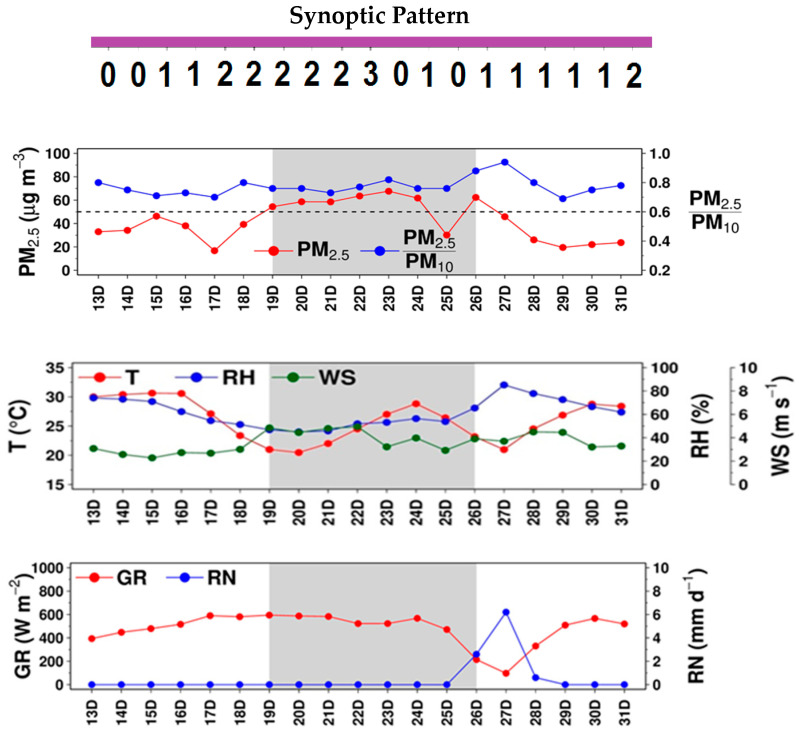
Daily PM_2.5_, PM_2.5_/PM_10_, and other meteorological variables during EP2.

This episode (EP2) occurred in December 2017, with pre-HZ2 on 13–18 December, the haze event (HZ2) on 19–26 December 2017 and post-HZ2 on 27–31 December. Daily PM_2.5_, PM_2.5_/PM_10_ ratio, temperature, relative humidity, wind speed, global radiation, rain, and synoptic pattern for EP2 are given in Figure 6. As seen, the average PM_2.5_ was less than 50 µg m^−3^ for all days during pre-HZ2. At the start of HZ2 on 19 December, PM_2.5_ increased to 54.4 µg m^−3^ and remains consistently higher than 50 µg m^−3^ until 26 December (except on 25 December). PM_2.5_ dropped sharply after 26 December due likely to wet scavenging by rain reported for three consecutive days (26–28 December) caused by a tropical depression in the Gulf of Thailand [57]. The tropical depression was the final phase of Typhoon Tembin maturely developed in the South China Sea. PM_2.5_/PM_10_ appeared to be higher in EP2 (0.69–0.94) than that in EP1 (0.45–0.66). The lower ratio in EP1 was due partly to significant contributions from agricultural burning and forest fires. Based on a global emission database by Klimont et al. (2017) [58], it was found that the PM_2.5_/PM_10_ ratio for emissions from agricultural burning and forest fires was lower as compared to those for other anthropogenic emissions. Thus, biomass burning observed in EP1 may have decreased the PM_2.5_/PM_10_ ratio. Haze episode EP2 did not show effects of biomass burning, which is explained in the latter part of this section. Temperature and relative humidity on 13 December was 30 °C and 74.1%, respectively and did not change much until 16 December. The synoptic change from Pattern 1 to Pattern 2 on 16–17 December, which caused an abrupt decrease in both variables until Pattern 2 prevailed, i.e., 20 December. Temperature and relative humidity dropped to 20.5 °C and 45.1%, respectively, on 20 December and gradually climbed up to 28.8 °C and 56.4%, respectively, on 24 December due to the weakening of cold surge leading to the return of the warm moist air. Once the cold surge recedes, Pattern 0 and Pattern 1 were dominant for most of the days. Global radiation increased from 393.8 W m^−2^ on 13 December to 594.7 W m^−2^ on 19 December (i.e., the start of HZ2) due to less cloud cover caused by the cold surge. Over 24–27 December, both temperature and global radiation declined and relative humidity increased, as influenced by the tropical depression. The tropical depression and rainfall observed during 26–28 December likely increased relative humidity during 24–27 December. The temperature rose after 27 December until the end of EP2 due to an increase in global radiation, which in turn decreased relative humidity. Global radiation was consistently higher during the haze event until 24 December with less variation and decreased later between 24 and 27 December and then again increased until the end of EP2 during 27–31 December. The wind followed the synoptic patterns quite closely. It was relatively strong during the active phase of cold surge during pre-HZ2 (particularly, 19–22 December) and starting four days of HZ2 while for the other days it was relatively weak.

The diurnality during HZ2 was clearer as compared to that during the pre-HZ2 and post-HZ2 (Figure S4 in Supplementary Materials). An obvious diurnal pattern with higher PM_2.5_ during the night and early morning and drop during the afternoon were observed during HZ2. Obukhov length indicated stability conditions for some hours during a few days. However, no clear change in stability was observed throughout the haze event before the tropical depression.

**Figure 7:**
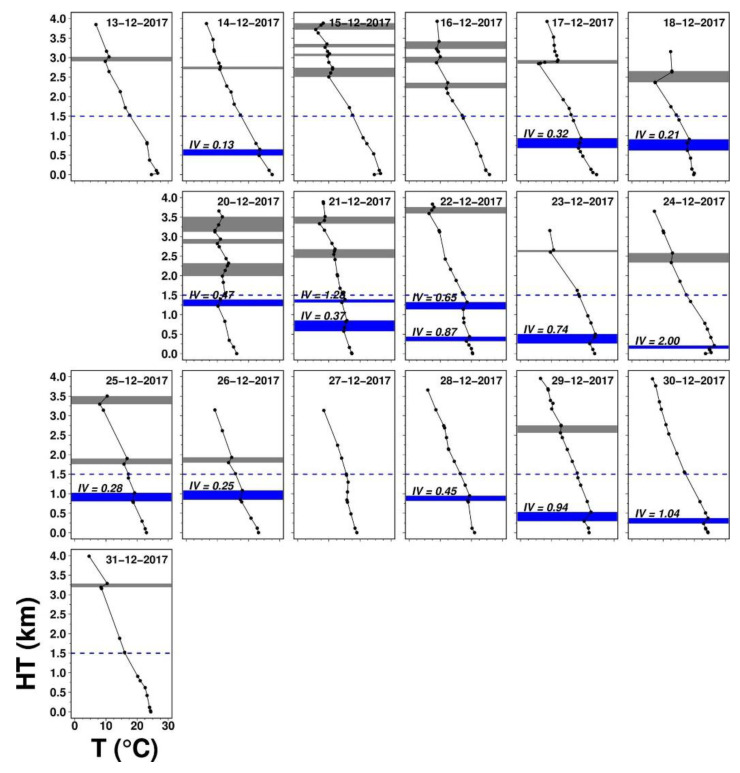
Same as Figure 4, but for haze episode EP2. *IV* represents the inversion intensity in °C/100 m.

The vertical temperature profiles with inversion layers for all days in this episode are shown in Figure 7. Three out of six days during pre-HZ2 do not show any temperature inversion while all non-missing seven days during HZ2 between 19 and 26 December show temperature inversions of varying intensity strength with a minimum of 0.25 °C/100 m on 26 December and a maximum of 2.0 °C/100 m on 24 December (Figure 7). Multiple low-level inversion layers were found during 21–22 December. 2 out of 5 days during post-HZ2 did not show any inversion. High-level inversions existed for most of the days during EP2 due to the presence of large-scale subsidence inversion aloft in the winter.

Lastly, we used back-trajectories from HYSPLIT to investigate the effect of long-range transport of air pollutants (Figure 5 and Figure S5 in Supplementary Materials). Except for 13–14 December, all the days had longer trajectories and coming from the eastern and northeastern directions. Trajectories during pre-HZ2 and post-HZ2 were mostly low level, while for HZ2, it was at a high level for many days (Figure S5 in Supplementary Materials). Fewer fire hot spots for all three periods: pre-HZ2, HZ2, and post-HZ2 were found in nearby regions suggesting that this haze episode HZ2 was mainly caused by the synergetic effect of local emission and local/synoptic meteorology (Figure S6 in Supplementary Materials). Comparing the two episodes, the EP2 back-trajectories tended to be longer (i.e., move faster). Fast-moving back-trajectories generally had short residential times to absorb atmospheric constituents. These imply limited contribution from biomass burning to haze, and elevated haze in this episode may have been more driven by local emissions.

It is noted that the contrasting results of the potential impact of biomass burning on the two episodes suggest that the influence of biomass burning may have varied by episode. Following our literature survey for Bangkok or Greater Bangkok (see Table S1 in Supplementary Materials), Phairuang et al. (2019) [33] found agricultural burning in the central region of Thailand and forest fires in remote areas (e.g., the northern region) to contribute to particulate matter in Bangkok, but the impact is more evident during the winter (November–January) when air masses are generally continental and favorably transporting haze to the study area, as compared to the summer (March) when air masses are relatively clean due to originating in or passing over the Gulf of Thailand. Similarly, Kayee et al. (2020) [35] looked into a summertime haze episode and found no significant contribution from biomass burning in the northern region due likely to lack of continental air masses. Dejchanchaiwong et al. (2020) [34] reported biomass burning in Thailand and Cambodia with favorable air masses as a major contributor to a wintertime haze episode.

## 4. Conclusions

Several local and synoptic meteorological aspects associated with the selected two wintertime haze episodes in Greater Bangkok were examined using observational data, including classified four synoptic patterns, day-to-day and diurnal variation, dynamic stability, temperature inversion, and back-trajectories. The first episode included an elevated haze event of 16 days (14–29 January 2015), together with some days before and after the haze event. However, the second episode had an elevated haze event of only 8 days (19–26 December 2017). Daily PM_2.5_ was found to be 50 µg m^−3^ or higher over most of each haze event. The two haze events commonly had cold surges as the background synoptic feature to initiate or trigger haze evolution. The cold surge reached the study area before the start of each haze event, causing temperature and relative humidity to drop abruptly initially but then to gradually increase as the cold surge weakened or dissipated. Wind speed was relatively large when the cold surge was active. Global radiation was generally modulated by cloud cover, which turns relatively high during each haze event because the cold surge induced fewer clouds. Daytime dynamic stability was generally unstable along the course of each haze event, except being stable at the ending of the second haze event due to a tropical depression. In each haze event, low-level temperature inversion existed well, with double layers seen in the beginning, effectively suppressing atmospheric dilution. Large-scale subsidence inversion aloft was also persistently present. In the first haze event, relatively low PM_2.5_ was observed in the afternoon over its first part, but a higher afternoon PM_2.5_ occurred over its second part, due possibly to the role of secondary aerosols. Comparing the two episodes, the PM_2.5_/PM_10_ ratio was relatively low in the first episode because of more impact of biomass burning, which was in general agreement with back-trajectories and active fire hotspots. The second haze event, with little biomass burning in the region, was likely to be caused mainly by local anthropogenic emissions.

According to the above findings, certain policy-related implications are given as follows:

(1) Local and synoptic meteorological factors play an important role in the elevated haze over Greater Bangkok. This poses a challenge to haze mitigation by emission reduction. Cold surge leads to an abrupt change in local meteorological conditions with less atmospheric ventilation as a result. A time window to apply control measures for haze is thus limited, emphasizing readiness in haze mitigation responses and early public warning with reliable weather/air quality forecasting.
(2) Apart from conventional anthropogenic emissions, biomass burning is another emission sector that potentially contributes to the urban haze in the study area. Thus, agricultural burning and forest fires need to be considered, and better controlled/managed in support of haze mitigation.
(3) It is fair to say that the contributing degrees of different emission sectors (i.e., anthropogenic and biomass burning) may vary by episode or over days within an episode and that secondary aerosols can as well form and partly contribute. Haze-related policymaking needs an integrated approach dealing with all important emission sectors for both particulate and gaseous precursors.

Future works on the urban haze problem in the study area may be extended to the following aspects: urban heat island, local recirculation, and systematic synoptic pattern classification [59], hourly mixing height variation and application of high-resolution light detection and ranging (LiDAR) if available [60], low-visibility events, and episode-specific source–receptor studies using source apportionment, chemical characterization (e.g., carbonaceous components in PM_2.5_), and air quality modeling.

## Figures and Tables

**Table 1:** Monitoring stations considered in this study.

Station	Province	Variables	Station Type (Background)
P27	Samut Sakhon	Hourly PM_2.5_, and PM_10_	Surface air quality (roadside or semi-general)
P59	Bangkok	Hourly PM_2.5_, and PM_10,_ *RH*	Surface air quality (semi-general)
P61	Bangkok	Hourly PM_2.5_ and PM_10_	Surface air quality (general)
M6	Pathum Thani	Hourly *T2*, *T50*, *WS50*, *WS100*, *WD50, WD100*, *RH*, *RN*, and *GR*	100-m tower (general)
TMD	Bangkok	*T* (at different heights)	Sounding at 7 LT

Note: PM_2.5_ and PM_10_ (µg m^−3^): particulate matter with size not larger than 2.5 µm and 10 µm, respectively; *T2* and *T50* (°C): temperature at 2 m and 50 m, respectively; *WS50* and *WS100* (m s^−1^): wind speed at 50 m and 100 m, respectively; *WD50* and *WD100* (degrees from the north): wind direction at 50 m and 100 m, respectively; *RH* (%): relative humidity; *RN* (mm): rain; *GR* (W m^−2^): global radiation; the stations have the terrain elevations of 2–4 m MSL.

## Supplementary Materials

The following are available online at https://www.mdpi.com/1660-4601/17/24/9499/s1. Table S1. Selected air pollution studies in Greater Bangkok. Figure S1. Daily PM_2.5_ variation at the three air quality stations by year: (a) 2015; (b) 2016; (c) 2017. Figure S2. Example surface charts in EP1 corresponding to the four synoptic patterns classified: (a) Pattern 0; (b) Pattern 1; (c) Pattern 2; (d) Pattern 3. Figure S3. Example surface charts in EP2 corresponding to the four synoptic patterns classified: (a) Pattern 0; (b) Pattern 1; (c) Pattern 2; (d) Pattern 3. Figure S4. Diurnal variation of PM_2.5_ and Obukhov length (*L*) during: (a) EP1 in 2015; (b) EP2 in 2017. Figure S5. Vertical migration of each individual daily 48-h back-trajectory: (a) EP1 in 2015; (b) EP2 in 2017. Figure S6. Average daily active fire hotspots per pixel (0.5° by 0.5°): (a) EP1 in 2015; (b) EP2 in 2017.
