# Supplementary material for: Evolution of Urban Haze in Greater Bangkok and Association with Local Meteorological and Synoptic Characteristics during Two Recent Haze Episodes
Source: Int J Environ Res Public Health. 2020 Dec 18;17(24):9499. doi: 10.3390/ijerph17249499 (PMC7766008; doi:10.3390/ijerph17249499)
Supplement: Supplementary file 1 [file ijerph-17-09499-s001.pdf]

International Journal of
Environmental Research and Public Health

Table S1. Selected air pollution studies in Greater Bangkok.

Study	Period	Key Findings
Source apportionment by Chuersuwan et al. (2008) [1]	February 2002–January 2003	Non-season specific: At roadside sites, traffics and biomass burning are two main sources of both PM_{10} and $PM_{2.5}$. At residential sites, same as the roadside sites for PM_{10} but biomass burning and as main sources for $PM_{2.5}$.
Source apportionment by Wimolwattanapun et al. (2011) [2]	January 2003–December 2007	Non-season specific: Traffic emission and biomass burning are the main sources of $PM_{2.5}$. Dust soil and construction soil are those of coarse PM mode.
Source identification by chemical characterization of carbonaceous aerosols by ChooChuay et al. (2020) [3]	August 2017–March 2018	Non-season specific: Traffic emission and biomass burning the main sources of $PM_{2.5}$.
Source apportionment by Narita et al. (2019) [4]	September 2015–March 2017	Traffic emission, biomass burning, and secondary inorganic aerosols are the main sources of $PM_{2.5}$ in both dry and wet seasons.
Source identification by analysis of carbonaceous aerosols by Phairuang et al. (2019) [5]	August 2014–July 2015	Carbonaceous aerosols in ultrafine and fine particulate matter significantly contributed by open burning of agricultural residues within the study area and from nearby and remote regions, especially in the winter and the summer but a shift in air mass trajectories lessens the biomass burning contribution in the latter season.
Source identification by analysis of carbonaceous aerosols by Dejchanchaiwong et al. (2020) [6]	2017–2019 (not continuous, only selected months)	Similar to Phairuang at al. (2019) [5] but with the potential long-range transport of smoke haze from Cambodia in the winter.
Source identification using trace metals by Kayee et al. (2020) [7]	January–April 2019	Industries, coal/fossil-fuel combustion, traffics, and sea spray are the main sources of dry-season $PM_{2.5}$. No significant contribution from biomass burning in the northern region was found.
Industrial and power-plant emission inventory in Thailand by Pham et al. (2008) [8]	2004	The central and eastern regions have relatively large emissions.
$PM_{2.5}$ health risk by Thongthammachart et al. (2020) [9]	January 2015–December 2017	Hazard quotient of $PM_{2.5}$ becomes higher during December-February than in the other times of the year.
$PM_{2.5}$ and annual mortality by Fold et al. (2020) [10]	January 2012–December 2018	Using BenMap-CE, premature mortality in Bangkok is reduced by about 2.3 times if the stricter annual $PM_{2.5}$ standard of the World Health Organization (WHO) are applied and complied with, as opposed to the Thai annual standard.

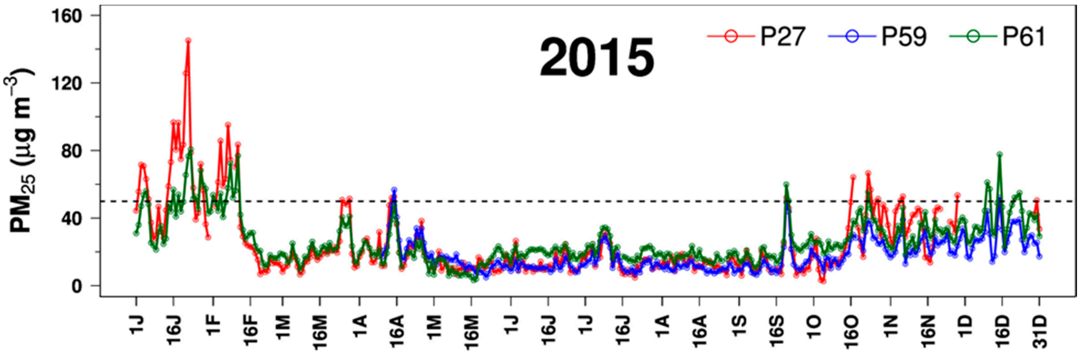

(a)

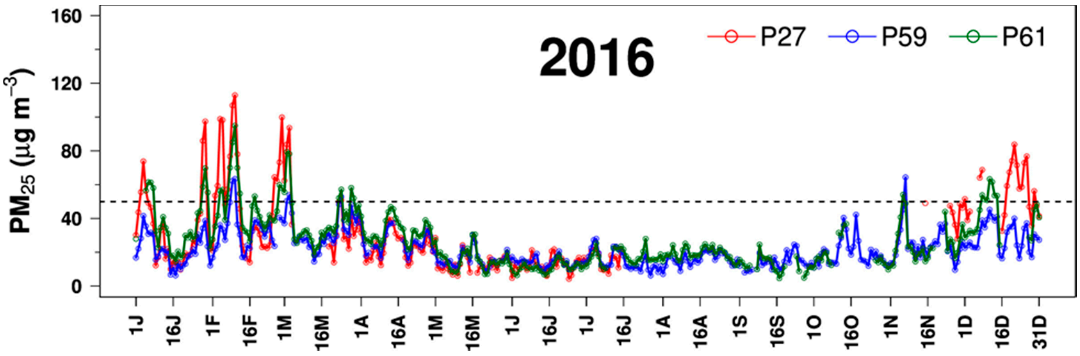

(b)

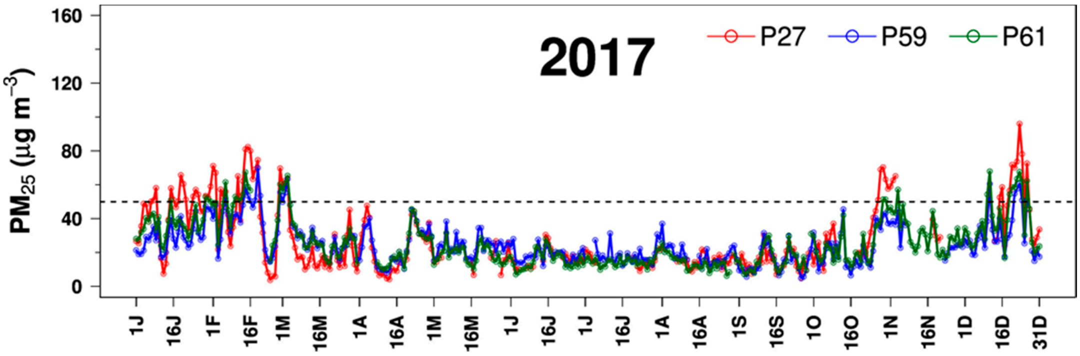

(c)

Figure S1. Daily $PM_{2.5}$ variation at the three air quality stations by year: (**a**) 2015; (**b**) 2016; (**c**) 2017. The *x*-axis labels (J, F, M, ..., N, and D) denote the months of year.

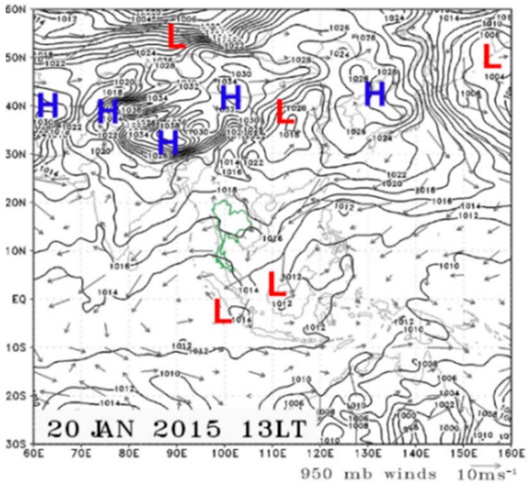

(a) Pattern 0

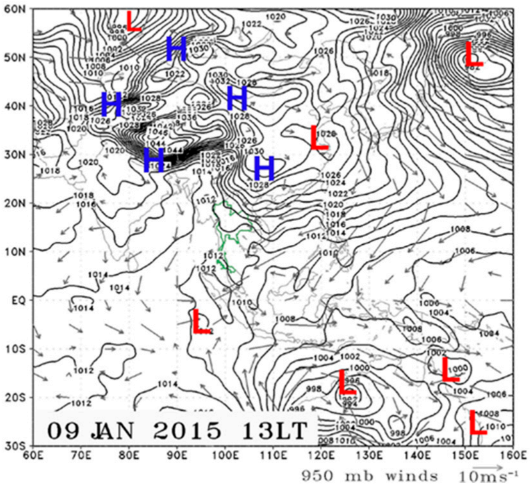

(b) Pattern 1

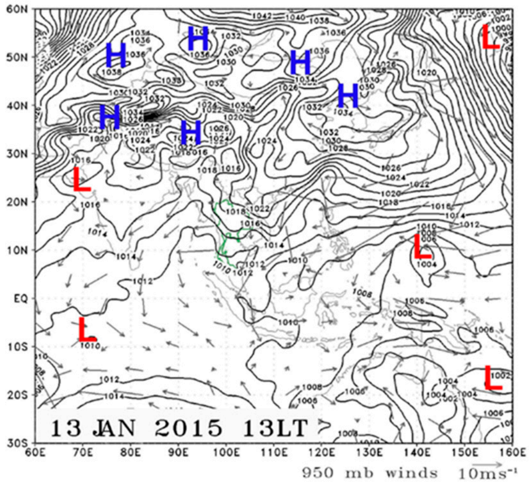

(c) Pattern 2

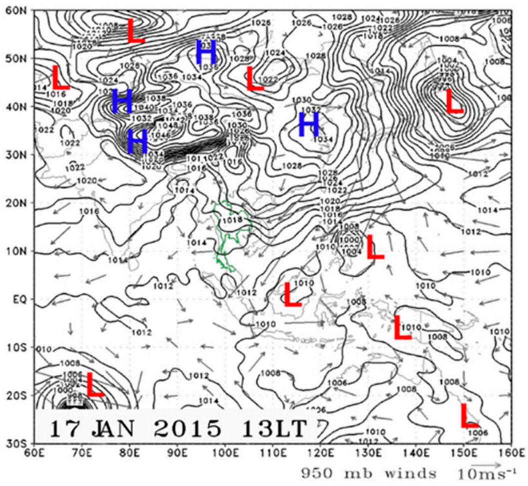

(d) Pattern 3

Figure S2. Example surface charts in EP1 corresponding to the four synoptic patterns classified: (**a**) Pattern 0; (**b**) Pattern 1; (**c**) Pattern 2; (**d**) Pattern 3.

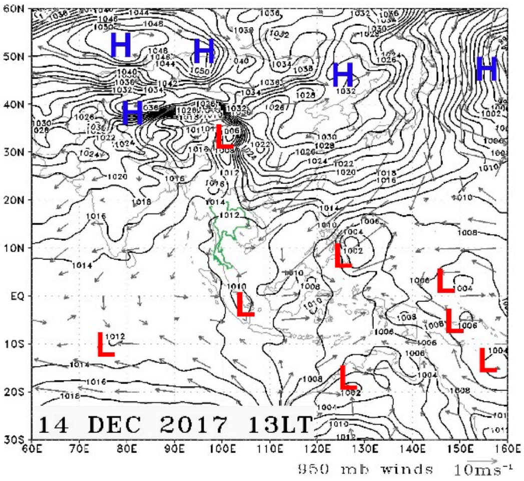

(a) Pattern 0

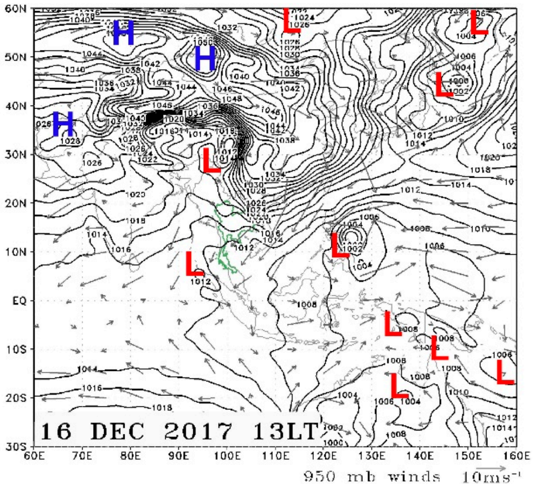

(b) Pattern 1

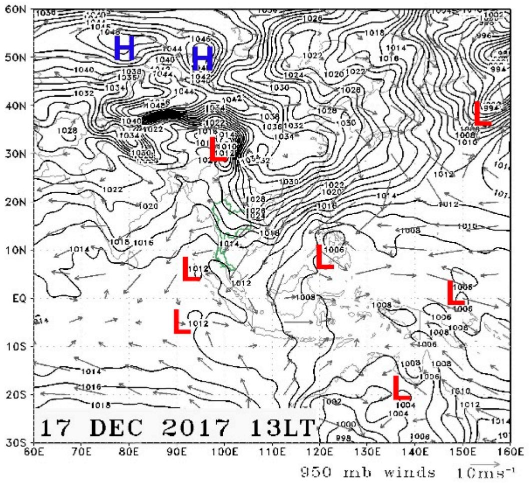

(c) Pattern 2

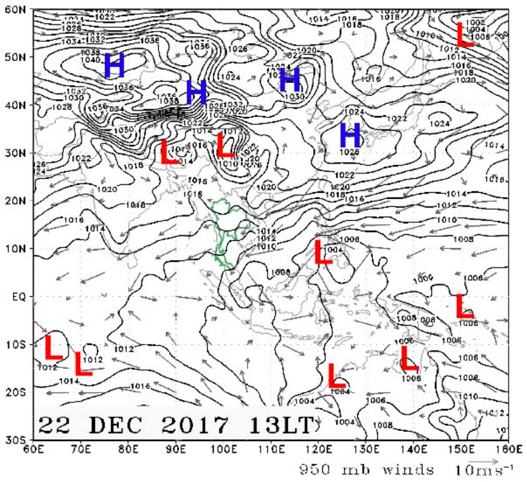

(d) Pattern 3

Figure S3. Example surface charts in EP2 corresponding to the four synoptic patterns classified: (**a**) Pattern 0; (**b**) Pattern 1; (**c**) Pattern 2; (**d**) Pattern 3.

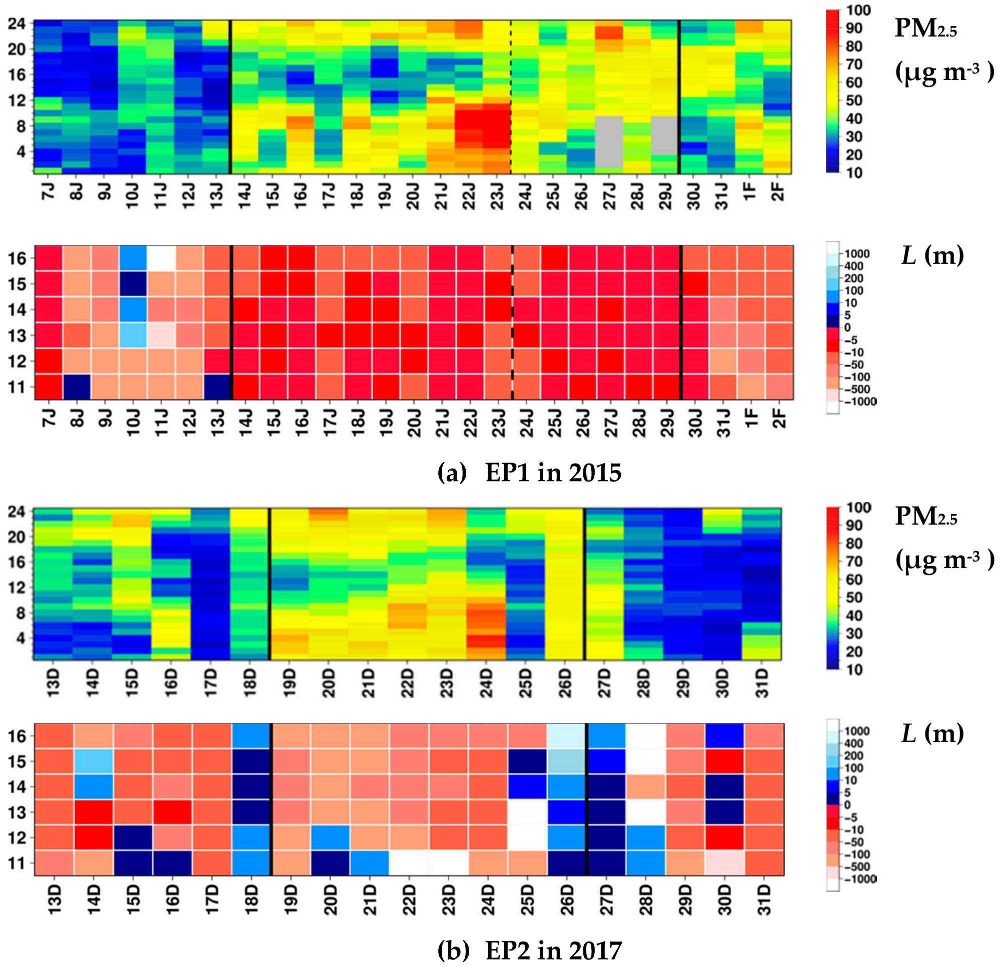

Figure S4. Diurnal variation of $PM_{2.5}$ and Obukhov length (L) during: (**a**) EP1 in 2015; (**b**) EP2 in 2017.

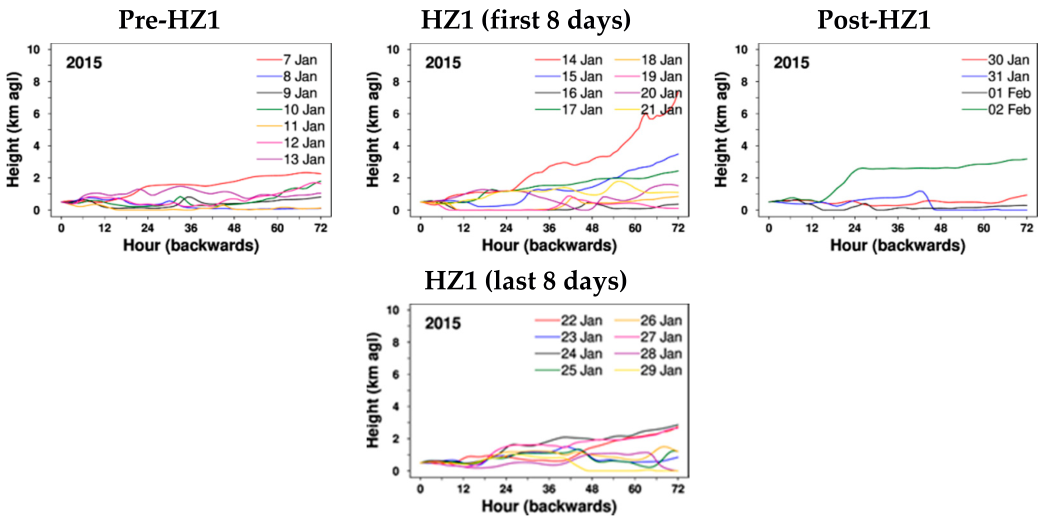

(a) EP1 in 2015

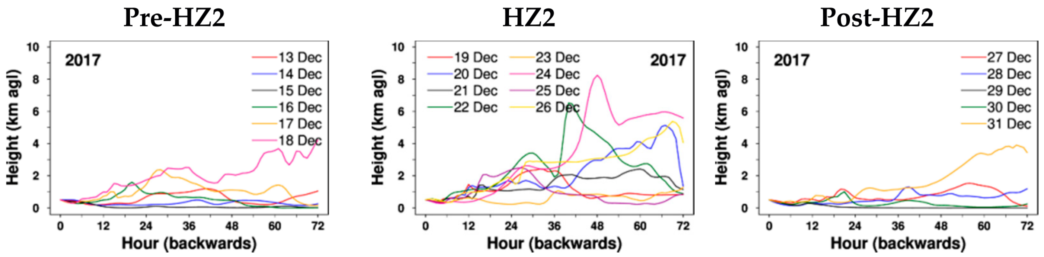

(a) EP2 in 2017

Figure S5. Vertical migration of each individual daily 48-h back-trajectory: (**a**) EP1 in 2015; (**b**) EP2 in 2017.

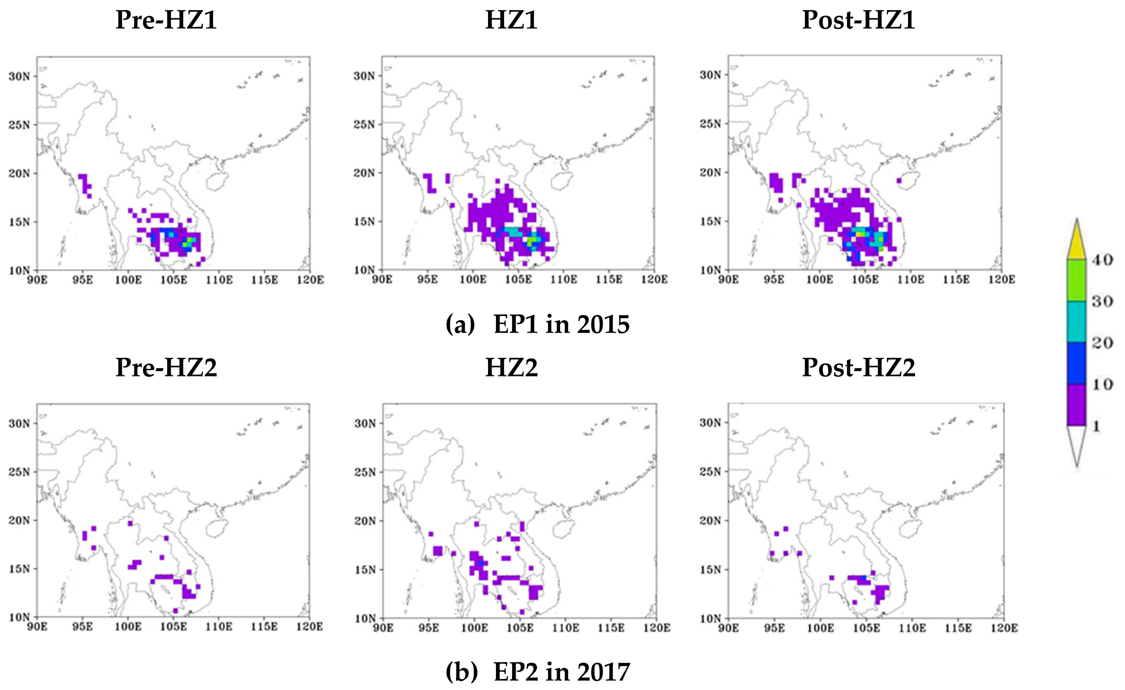

Figure S6. Average daily active fire hotspots per pixel (0.5° by 0.5°): (**a**) EP1 in 2015; (**b**). EP2 in 2017.

References

1. Chuersuwan, N.; Nimrat, S.; Lekphet, S.; Kerdkumrai, T. Levels and major sources of $PM_{2.5}$ and PM_{10} in Bangkok Metropolitan Region. *Environ. Int.* **2008**, *34*, 671–677.
2. Wimolwattanapun, W.; Hopke, P.K.; Pongkiatkul, P. Source apportionment and potential source locations of $PM_{2.5}$ and $PM_{2.5\text{-}10}$ at residential sites in metropolitan Bangkok. *Atmos. Pollut. Res.* **2011**, *2*, 172–181.
3. ChooChuay, C.; Pongpiachan, S.; Tipmanee, D.; Suttinun, O.; Deelaman, W.; Wang, Q.; Xing, Li.; Li, G.; Han, Y.; Palakun, J.; Cao, J. Impacts of $PM_{2.5}$ sources on variations in particulate chemical compounds in ambient air of Bangkok, Thailand. *Atmos. Pollut. Res.* **2020**, *11*, 1657–1667.
4. Narita, D.; Oanh, N.T.K.; Sato, K.; Huo, M.; Permadi, D.A.; Chi, N.N.H.; Ratanajaratroj, T.; Pawarmart, I. Pollution characteristics and policy actions on fine particulate matter in a growing Asian economy: The case of Bangkok Metropolitan Region. *Atmosphere* **2019**, *10*, 227.
5. Phairuang, W.; Suwattiga, P.; Chetiyanukornkul, T.; Hongtieab, S.; Limpaseni, W.; Ikemori, F.; Hata, M.; Furuuchi, M. The influence of the open burning of agricultural biomass and forest fires in Thailand on the carbonaceous components in size-fractionated particles. *Environ. Pollut.* **2019**, *247*, 238–247.
6. Dejchanchaiwong, R.; Tekasakul, P.; Tekasakul, S.; Phairuang, W.; Nim, N.; Sresawasd, C.; Thongboon, K.; Thongyen, T.; Suwattiga, P. Impact of transport of fine and ultrafine particles from open biomass burning on air quality during 2019 Bangkok haze episode. *J. Environ. Sci. (China)* **2020**, 97, 238–247.
7. Kayee, J.; Sompongchaiyakul, P.; Sanwlani, N.; Bureekul, S.; Wang, X.; Das, R. Metal concentrations and source apportionment of $PM_{2.5}$ in Chiang Rai and Bangkok, Thailand during a biomass burning season. *ACS Earth Space Chem.* **2020**, *4*, 1213–1226.
8. Pham, T.B.T.; Manomaiphiboon, K.; Vongmahadlek, C. Development of an inventory and temporal allocation profiles of emissions from power plants and industrial facilities in Thailand. *Sci. Total Environ.* **2008**, *397*, 103–118.
9. Thongthammachart, T.; Jinsart, W. Estimating $PM_{2.5}$ concentrations with statistical distribution techniques for health risk assessment in Bangkok. *Hum. Ecol. Risk Assess*. **2020**, *26*, 1848–1863.
10. Fold, N.R.; Allison, M.R.; Wood, B.C.; Pham T. B. T.; Bonnet, S.; Garivait, S.; Kamens, R.; Pengjan, S. An assessment of annual mortality attributable to ambient $PM_{2.5}$ in Bangkok, Thailand. *Int. J. Environ. Res. Public Health* **2020**, *17*, 7298.
